# Detection of viable and total fungal community in zaopei of Chinese strong-flavor baijiu using PMA combined with qPCR and HTS based on ITS2 region

**DOI:** 10.1186/s12866-021-02334-8

**Published:** 2021-10-08

**Authors:** Huanming Liu, Guangxun Tan, Qitong Chen, Weiwei Dong, Ping Chen, Kaiyun Cai, Yuanliang Hu, Weiyan Zhang, Nan Peng, Yunxiang Liang, Shumiao Zhao

**Affiliations:** 1 State Key Laboratory of Agricultural Microbiology and College of Life Science and Technology, Huazhong Agricultural University, Wuhan, 430070 China; 2 School of Food Science and Technology, Guangdong Ocean University, Zhanjiang, 524088 China; 3 Hubei Daohuaxiang Liquor Co., Ltd, Yichang, 443112 China; 4 Hubei Key Laboratory of Edible Wild Plants Conservation & Utilization, College of Life Sciences, Hubei Normal University, Huangshi, 435000 China; 5 Li Dak Sum Yip Yio Chin Kenneth Li Marine Biopharmaceutical Research Center, Ningbo University, Ningbo, 315211 China

**Keywords:** Chinese strong-flavor baijiu, Zaopei, PMA, Viable fungal community

## Abstract

**Background:**

Chinese strong-flavor baijiu (CSFB), one of the three major baijiu types, is the most popular baijiu type among consumers in China. A variety of microbes are involved in metabolizing raw materials to produce ethanol and flavor substances during fermentation, which fundamentally determined the quality of baijiu. It is of great importance to study microbial community of fermented grains (zaopei) during baijiu brewing process for improving its quality. In this study, we firstly used propidium monoazide (PMA) to treat zaopei samples from 5-year pit and 20-year pit for removing the interference of non-viable fungi, and analyzed the diversity of total fungi and viable fungi by quantitative PCR (qPCR) and high-throughput sequencing (HTS) based on ITS2 gene.

**Results:**

The results showed that total fungi and viable fungi displayed no significant differences at OTU, phylum, or genus levels during fermentation within two kinds of pits. A total of 6 phyla, 19 classes, and 118 genera in fungi were found based on OTUs annotation in zaopei samples from 5-year pit and 20-year pit. Besides, non-viable fungi had little effect on the fungal community diversity during the fermentation cycle. It was found that the most dominant viable fungi belonged to *Saccharomyces*, *Kazachstania*, *Naumovozyma*, and *Trichosporon*, and *Naumovozyma* was firstly detected in zaopei samples of CSFB. Moreover, based on the variation of flavor substances in zaopei samples, the quality of CSFB produced from older pit was better than that produced from younger pit.

**Conclusion:**

The non-viable fungi had little effect on the fungal diversity, structure, and relative abundance in zaopei samples of CSFB, and *Naumovozyma* was firstly detected in zaopei samples of CSFB. Our findings can be applied as guidance for improving the quality and stability of CSFB.

**Supplementary Information:**

The online version contains supplementary material available at 10.1186/s12866-021-02334-8.

## Introduction

**Fig. 1:**
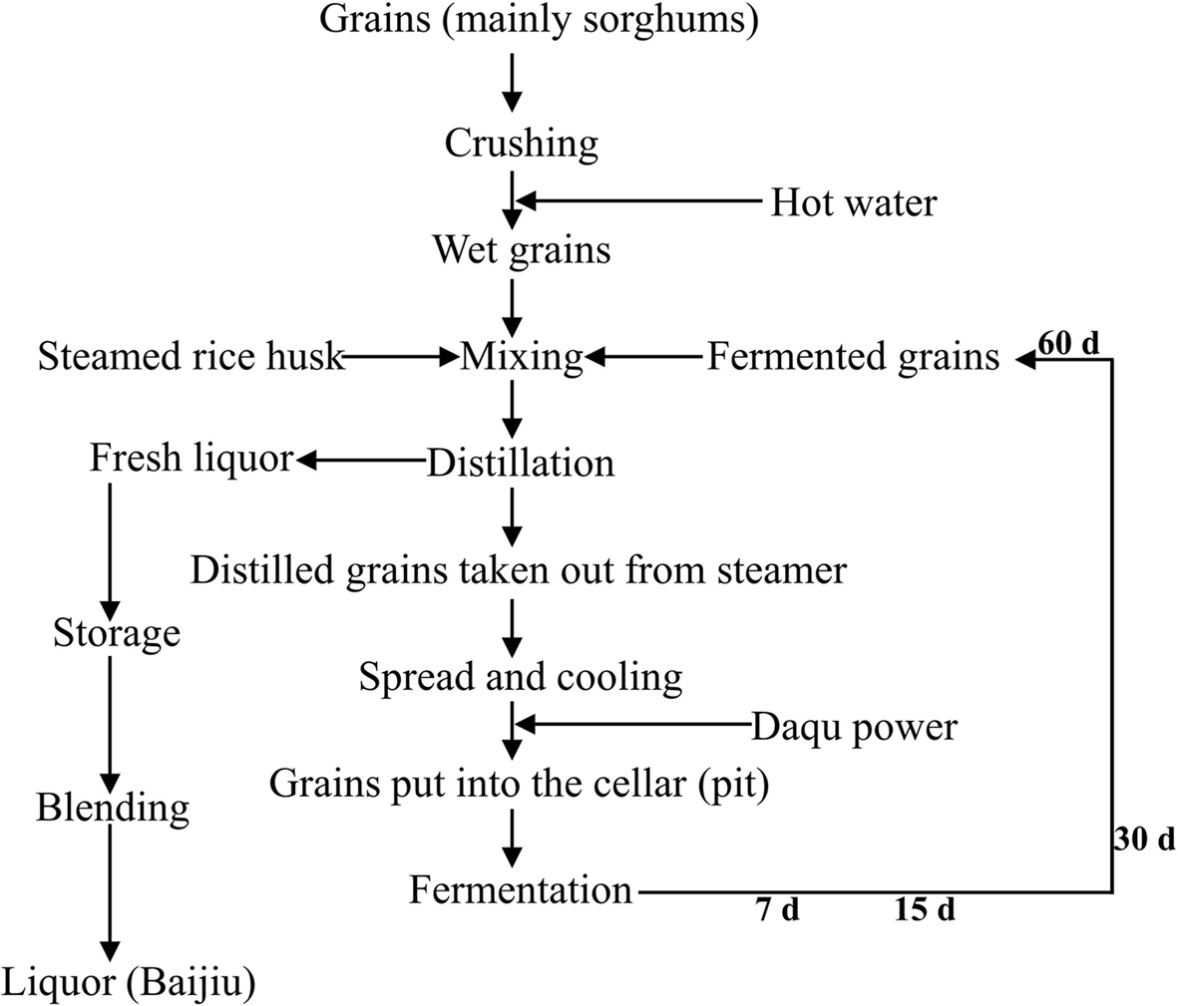
The fermentation process of Chinese strong-flavor baijiu (CSFB)

Chinese baijiu, a historic alcoholic beverage, is distilled from spontaneously solid-state fermented grains. Baijiu is divided into three major types, namely sauce-flavor, strong-flavor, and light-flavor. Because of the fragrant flavor, soft mouthfeel, and long-lasting aftertaste in Chinese strong-flavor baijiu (CSFB) [1], it is very popular among consumers, and its production is the highest in the overall baijiu market, accounting for 70% of total baijiu production. Here, the sorghums and a small number of wheats and rice are raw material (grains). The grains are processed with many pre-procedures, and mixed with daqu powder (fermentation starter), then fermented in a special cellar called pit (Fig. 1). After distillation of the zaopei (fermented grains), ethanol, flavor compounds and water are collected together for storage and blending to produce final product-CSFB (Fig. 1). Many microorganisms from daqu and pit mud are involved in the solid-state fermentation, during which nutrient substances in grains were consumed for quick proliferation and producing ethanol and flavor compounds [1–3]. Organic acids and esters are the main flavor compounds, which play a key role on the quality of CSFB [2, 4]. Therefore, understanding the composition and change of the microbial community in zaopei was very critical for optimizing fermentation processes and improving the quality of the CSFB.

In recent years, lots of studies have investigated the change of microbial community in zaopei during the fermentation cycle. Two kinds of DNA-based molecular technologies, encompassing polymerase chain reaction-denaturing gradient gel electrophoresis (PCR-DGGE) and high-throughput sequencing (HTS), were mainly applied to analyze microbial consortia. The PCR-DGGE technology has been utilized to analyze the microbial constituent of zaopei from CSFB [5, 6], sauce-flavor baijiu [7–9], and light-flavor baijiu [10]. Meanwhile, the HTS technology has been widely used to characterize microbial community of zaopei from CSFB [11, 12], light-flavor baijiu [13, 14], and sauce-flavor baijiu [15]. However, these technologies ignored the DNA from the non-viable microbes when total DNA extraction, and the extracted total DNA contained DNA from the viable microbes and non-viable microbes. Accordingly, these studies only focused on analyzing the total microbial community, and neglected the influence of non-viable microbes on the microbial community structure, which making the results of microbial community inaccuracy.

In fact, microbes keep growing, proliferating, and dying constantly during the entire fermentation process of baijiu production. The dead microbes are those with damaged cell membranes and lost their cell function, termed as non-viable microbes [16]. Many researches have proved that the DNA from non-viable microbes can exist for a long time in their environment, so that they can be extracted and used as template for PCR amplification, which influenced the assessment of microbial community structure [17–20]. Considering that there were lots of non-viable microbes existing and remaining in zaopei during baijiu fermentation. It is necessary to evaluate the influence of non-viable microbes on variation and composition of microbial community in zaopei.

Propidium monoazide (PMA) is a photo-reactive dye that can modify DNA and prohibit PCR amplification [19]. Meanwhile, PMA is cell membrane-impermeable, so that it can’t penetrate the intact cell membrane of viable cells, and it can only entre the non-viable cells by penetrating their damaged cell membranes [19]. Thus, the genomic DNA from viable microbes can’t be bound by PMA, and only the genomic DNA from non-viable microbes can be bound by PMA. Based on these characteristics, the collected samples can be treated with PMA prior to DNA extraction, inhibiting the subsequent PCR amplification of the DNA from non-viable microbes (Fig. S1). Therefore, these unique features of PMA make it highly useful in selective detection of viable microbes in complex samples [21–24].

In this study, the zaopei samples were collected, treated with PMA, and followed with DNA extraction. The effect of non-viable fungi on fungal flora in zaopei samples can be eliminated based on application of quantitative PCR (qPCR) and HTS aiming at ITS2 gene. Thus, the differences in characteristic and variation between viable fungal community and total fungal community were accurately analyzed in zaopei samples during the fermentation process of CSFB, which will expand our knowledge of baijiu brewing.

## Materials and methods

### Brewing processes and zaopei sampling

The Liquor factory in Yichang city (Hubei Province, China) is a famous manufacturer to produce CSFB in Hubei province. The traditional manufacture processes were reported by Jin et al. (Fig. 1) [3]. Namely, (a) grains (mainly sorghum) were smashed, soaked in hot water, and then steamed, (b) daqu powders were added after distilled grains cooling, and the mixtures were put into the pit (a special cellar below ground) for fermentation with a period of 60 days, (c) the mixtures were spade out for distillation to collect fresh liquor, (d) the fresh liquor was stored and then blended for the last products.

**Fig. 2:**
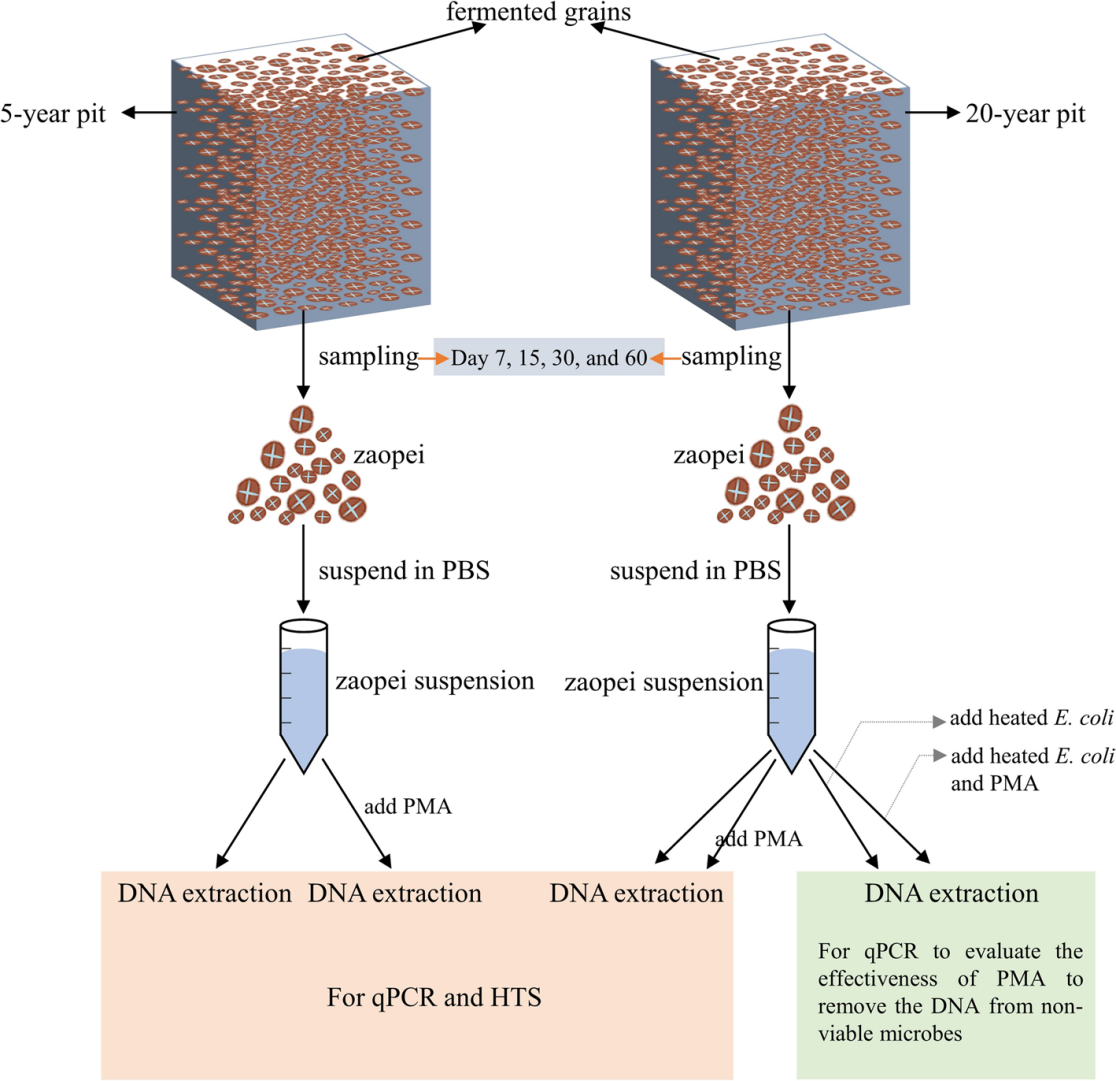
The schematic diagram of pre-treatments of zaopei samples before qPCR and HTS. The zaopei samples (*n* = 4) were collected at day 7, 15, 30, and 60 of the fermentation from two kinds of pits (5-year and 20-year). Samples were treated with PMA or not, and added the heated *E. coli* or not, then DNA extraction was performed

Two kinds of pits were used for producing baijiu in the Liquor factory, including 20-year pits and 5-year pits. Four 20-year pits and four 5-year pits were randomly chosen for sampling. The fermentation grains (zaopei) were collected from five positions distributed on three levels in each pit, including surface, middle, and bottom layer, mixed and then combined as one zaopei sample. The zaopei samples were collected from two kinds pits (20-year and 5-year) during different fermentation time at day 7, 15, 30, and 60 (Fig. 2). Herein, 32 zaopei samples were gathered for this study. Afterward, a total of 2.5 g zaopei sample was added in 100 mL 10 mM PBS (pH 7.4) to formulate the zaopei suspension for the further study.

### Physicochemical properties and flavor compounds analysis

The basic physicochemical properties of zaopei, including moisture content and acidity, were measured according to the methods of our previous study [14]. Moisture content was measured by weight loss after drying 10 g sample to constant weight. Acidity was detected by acid base titration with 10 g sample. Five grams sample was used for detecting starch content, which was based on the methods described by Yan’s study [25]. The content of lactic acid, butyric acid, acetic acid, ethyl acetate, ethyl caproate and ethyl lactate were detected by the methods depicted in our previous study [26].

### Positive control preparation and PMA treatment

The non-viable *Escherichia coli* (*E. coli*) cells were obtained by heating culture at 95 °C for 30 min to kill them. After centrifuging 400 μL heated culture at 10,000 rpm for 5 min, non-viable *E. coli* cells were suspended with 1 mL zaopei suspension to form a positive control sample. The positive control was designed to evaluate the PMA efficiency in each batch of PMA treatment. Given that there are more non-viable microorganisms in zaopei sampled in older pit than that in younger pit, and only zaopei samples collected in 20-year pit were used to prepare positive control. Therefore, the zaopei samples from 20-year pit, zaopei samples from 5-year pit and positive control were treated with 50 μM PMA according to the method of Tan et al. [16]. At last, zaopei suspension treated with PMA, positive control, and positive control treated with PMA were frozen at − 20 °C for DNA extraction.

### DNA extraction

The zaopei suspension from 20-year pit, zaopei suspension from 5-year pit, zaopei suspension from 20-year pit treated with PMA, zaopei suspension from 5-year pit treated with PMA, positive control, and positive control treated with PMA were used for DNA extraction (Fig. 2). The Fast DNA® SPIN Kit for Soil (MP Biomedicals, USA) was utilized to extract DNA, and the extracted DNA was stored at − 20 °C before further use. The concentration of DNA was detected by NanoDrop 2000 UV (Thermo, USA), and the quality of DNA was evaluated through 1% agarose gel electrophoresis.

### Quantitative PCR

The UNICON® qPCR SYBR® Green Master Mix (Yeasen, China) was used to amplify the ITS2 region of fungi within the ABI StepOne Plus qPCR instrument (Applied Biosystems, USA). To be specific, 20 μL qPCR mixture solution consisted of 10 μL Mix, 0.5 μL of forward primer, 0.5 μL of reverse primer, 2 μL of DNA template, and 7 μL of distilled water. Then amplification procedure was set as follow: 95 °C for 5 min, 95 °C for 5 s, 56 °C for 20 s, and 72 °C for 20 s for 40 cycles. Standard curves were developed according to our previous study [16], and the amplification efficiency was 95.0%. All the qPCR reactions were carried out in triplicate.

To evaluate the effectiveness of PMA to remove the DNA from non-viable microorganisms, the qPCR was carried out to amplify the V4 region of 16S rDNA with 515F and 806R primers [12]. The DNA template from positive control was utilized in qPCR for detecting the total bacteria, and the DNA template from positive control after PMA treatment was utilized in qPCR for detecting the viable bacteria. After qPCR, the difference of 16S rDNA copy number between PMA-treated positive control and untreated was analyzed by Rank sum test.

As for determining the content of fungi, the fITS7 and ITS4 primers were used to amplify the ITS2 region of fungal rRNA gene [27]. The DNA templates from zaopei suspension derived from two kinds of pits were utilized in qPCR for detecting total fungi, and the DNA templates from zaopei suspension derived from two kinds of pits treated with PMA were utilized in qPCR for detecting viable fungi. The Mann-Whitney U test was performed to analyze the difference in the ITS2 gene copy number between zaopei suspension.

### HTS and data analysis

The DNA was extracted from 64 samples, consisting of PMA-treated and untreated, taken from 2 kinds of pits (four 20-year pits and four 5-year pits) during 4 fermentation time (7 d, 15 d, 30 d, and 60 d). Then 10 ng of DNA extracted from each sample was used for PCR. For eukaryotes, the ITS2 region of fungal rDNA was amplified with primers fITS7 and ITS4 [27]. The PCR amplification was performed as follow: 94 °C for 3 min, 94 °C for 5 s, 57 °C for 60 s, and 72 °C for 20 s for 30 cycles, and final at 72 °C for 120 s. After purification, the PCR products were sequenced by Illumina MiSeq platform to generate 2 × 300 bp paired-end reads.

The raw sequencing data was processed by Pandaseq [28]. After sequence sort, sequence filter, and sequence removal [12], the rest sequences were clustered into operational taxonomic unit (OTU) with a sequence identity of 97% by Usearch (version 7.1). Then the representative sequence of each fungal OTU was extracted by QIIME and annotated by UNITE database. Afterward, the relative abundances at phylum, class and genus levels of each sample were computed based on the OTU annotations. The alpha diversity was evaluated by QIIME, and the principal coordinates analysis (PCoA) was conducted by in-house tool. The differential tests between viable and total fungal communities in the relative abundance of OTU and the Shannon index were performed by STAMP software and R package (Version 3.2).

### Data availability

Raw sequences generated by Illumina MiSeq sequencing in the present study have been deposited to NCBI with Bio Project accession number PRJNA746066.

## Results

### Physicochemical properties and flavor compounds analysis

**Fig. 3:**
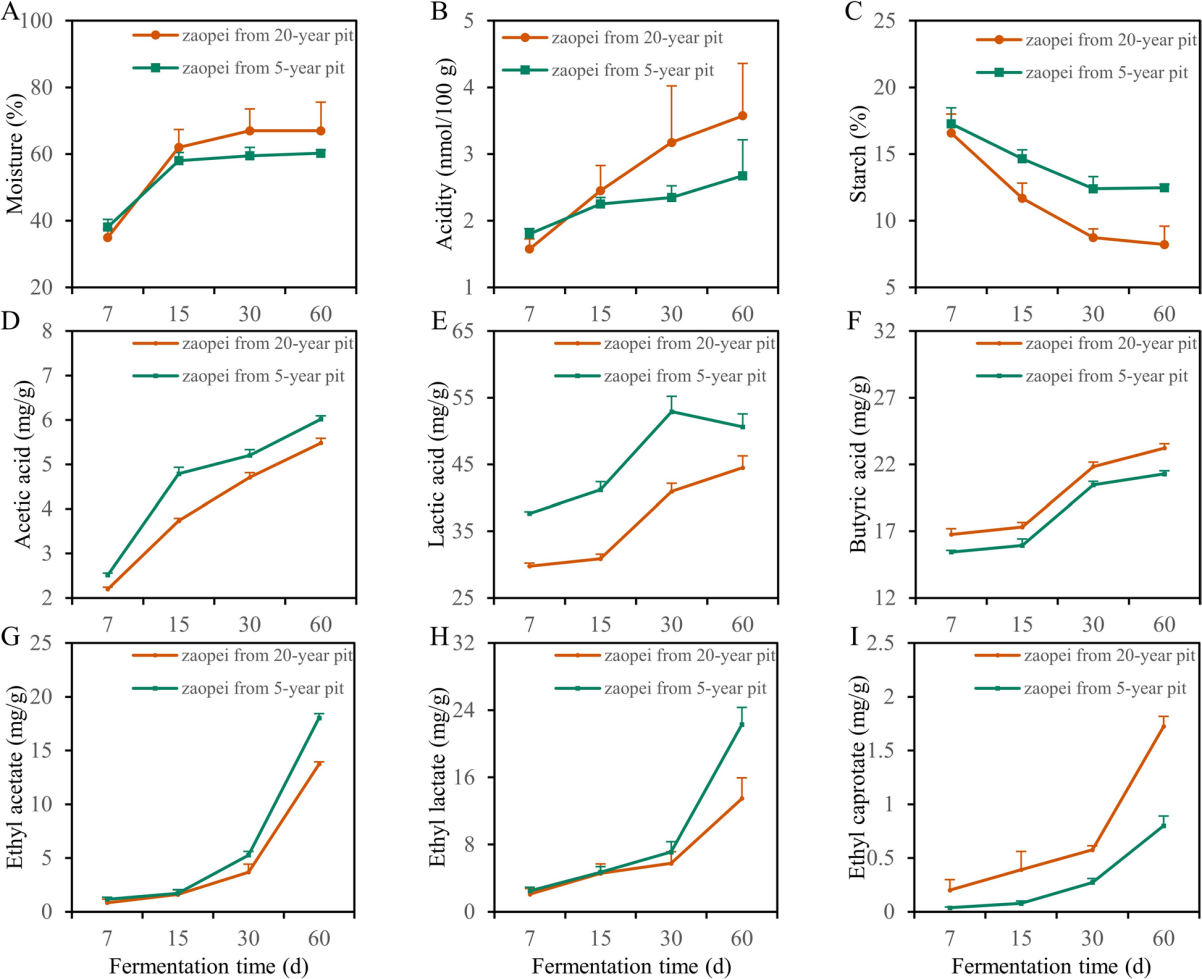
Changes of the content of moisture (**A**), acidity (**B**), starch (**C**), acetic acid (**D**), lactic acid (**E**), butyric acid (**F**), ethyl acetate (**G**), ethyl lactate (**H**) and ethyl caproate (**I**) in zaopei samples during fermentation. The one-way ANOVA test was used to compare the differences between two pits

The moisture content of zaopei from 20-year pit and 5-year pit showed a similar trend that increased from day 7 to day 30 and then kept stable until day 60 (Fig. 3A). Meanwhile, the moisture content of zaopei from 20-year pit was higher than that from 5-year pit except the day 7 (Fig. 3A). When considering the acidity, a same tendency was shared in zaopei samples from these two kinds of pits, which kept increasing from day 7 to day 60 (Fig. 3B). However, the acidity of zaopei from 20-year pit was higher than that from 5-year pit except the day 7 (Fig. 3B). As for the change of starch content, it decreased from day 7 to day 30 then stabilized from day 30 to day 60, and the starch content of zaopei from 20-year pit was lower than that of zaopei from 5-year pit during the entire fermentation (Fig. 3C).

The flavor compounds in zaopei played a key influence on the quality of baijiu, therefore, the content of acetic acid, lactic acid, butyric acid, ethyl acetate, ethyl lactate and ethyl caproate were detected during the total fermentation cycle. The content of acetic acid kept increase from day 7 to day 60 between these two kinds zaopei, and the content of acetic acid in zaopei from 5-year pit was higher than that from 20-year pit (Fig. 3D). For the content of lactic acid, it remained increase from day 7 to day 60 in zaopei from 20-year pit, while it increased from day 7 to day 30 then slightly decreased in zaopei from 5-year pit (Fig. 3E). Moreover, the lactic acid content of zaopei from 5-year pit was higher than that from 20-year pit (Fig. 3E). For the content of butyric acid, it exhibited a rise tendency in both zaopei samples from two kinds of pits (Fig. 3F). Besides, the content of butyric acid in zaopei from 20-year pit was higher than that from 5-year pit (Fig. 3F). Accordingly, the ester content in zaopei from two kinds pits displayed the similar results, and they all showed an increase trend (Fig. 3G, H, and I). Meanwhile, the contents of ethyl acetate and ethyl lactate in zaopei from 5-year pit was higher than those from 20-year pit, however, the content of ethyl caproate in zaopei from 20-year pit was higher than that from 5-year pit (Fig. 3G, H, and I).

### The effectiveness of PMA treatment

**Fig. 4:**
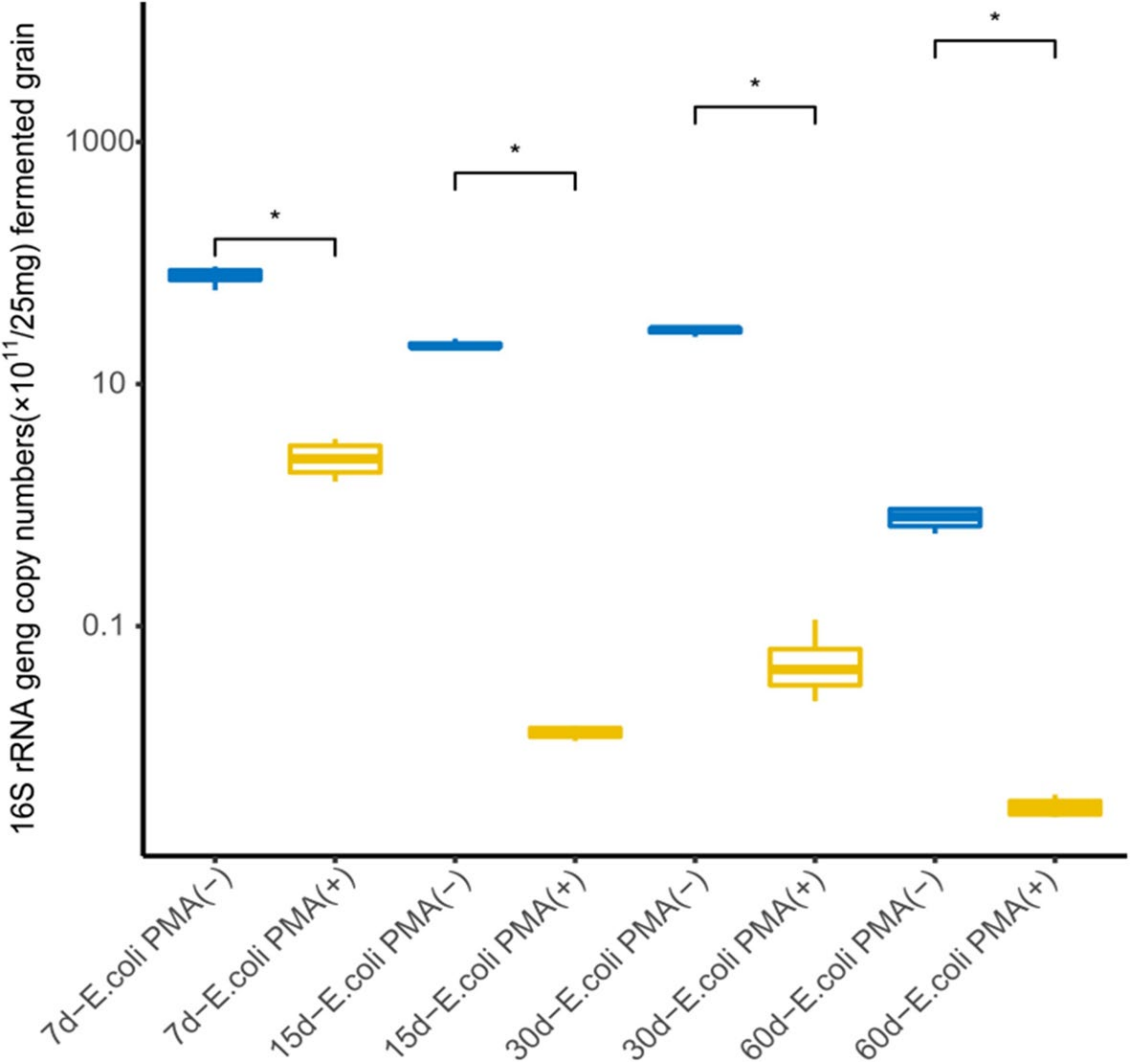
The effectiveness of PMA to remove the dead bacteria DNA in zaopei with adding heated *E. coli* cells. The zaopei samples (n = 4) were collected on the 7th, 15th, 30th and 60th day of fermentation (7 d, 15 d, 30 d, and 60 d) from 20-year pit. *E. coli* PMA (−): the positive control (dead *E. coli* cells and 1 mL zaopei suspension) was untreated with PMA; *E. coli* PMA (+): the positive control was treated with PMA. The Rank sum test was used to calculate the significant difference by R software (* represents significant difference)

There was 1 mL zaopei suspension (containing 25 mg zaopei) and heat-killed *E. coli* cells in each positive control sample. Herein, the DNA extracted from positive control treated with PMA was used for quantitating the viable bacteria, and the DNA extracted from positive control was used for quantitating the total bacteria. As shown in Fig. 4, the copy numbers of 16S rRNA gene from the positive control with PMA treatment were significantly lower than that without PMA treatment, reflecting a descend range during different fermentation time, with the value of 96.8, 99.9, 99.8, and 99.6%, respectively. Therefore, 50 μM PMA can effectively remove the interference of DNA from non-viable microbe cells with the number varying from 7.85 × 10^10^ to 2.11 × 10^13^.

### Quantification of total and viable fungi in zaopei

**Fig. 5:**
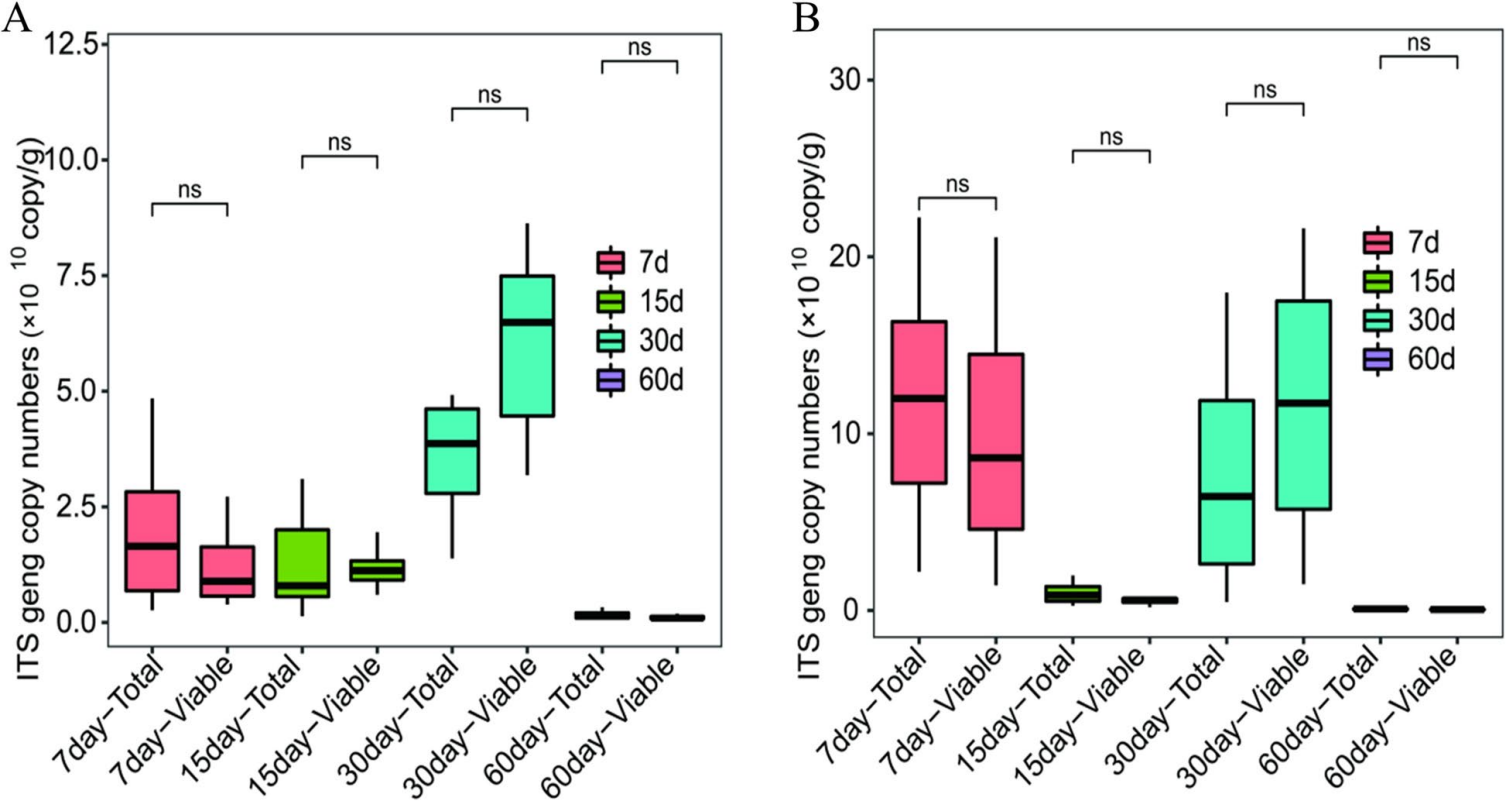
The qPCR analysis on total fungi and viable fungi in zaopei samples from pits with age of 5-year (**A**) and 20-year (**B**). The zaopei samples were treated with PMA for identifying viable fungi (Viable), whereas they were untreated with PMA for identifying total fungi (Total). The Rank sum test was used to calculate the significant difference by R software (* represents significant difference, ns represents no significant difference)

As depicted in Fig. 5, the copy numbers of ITS2 region gene in total fungi ranged from 3.20 × 10^6^ to 1.77 × 10^9^ in 25 mg zaopei from 5-year pit, while the copy numbers of ITS2 region gene in the total fungi varied from 6.35 × 10^5^ to 2.22 × 10^9^ in 25 mg zaopei from 20-year pit. Furthermore, the copy numbers of ITS2 region gene in the viable fungi ranged from 1.02 × 10^7^ to 8.73 × 10^8^ in 25 mg zaopei from 5-year pit, and the copy numbers of ITS2 region gene in the viable fungi varied from 5.00 × 10^6^ to 1.94 × 10^9^ in 25 mg zaopei from 20-year pit (Fig. S2). From these results, we can speculate that there were more fungi in zaopei from 20-year pit than that from 5-year pit. Moreover, there was no difference in total fungi and viable fungi between zaopei from 5-year pit and zaopei from 20-year pit during the whole fermentation cycle. The viable fungi accounted for 98 and 97% of total fungi in zaopei from 5-year pit and zaopei from 20-year pit (data not shown), respectively, indicating that there were little non-viable fungi during the fermentation process.

### Diversity and structural analysis of total and viable fungi in zaopei

After HTS of ITS2 gene in 64 zaopei samples from two kinds of pits with/without PMA treatment, there were totally 3,020,157 high-quality reads (47,189 on average) with an average length 320 bp (Table S1). A total of 223 OTUs were grouped in ITS2 sequences from all samples. After rarefaction, it showed a clear asymptote reflecting a near complete sampling of the fungal community. A total of 6 phyla, 19 classes, and 118 genera in fungi were identified based on OTUs annotation.

**Fig. 6:**
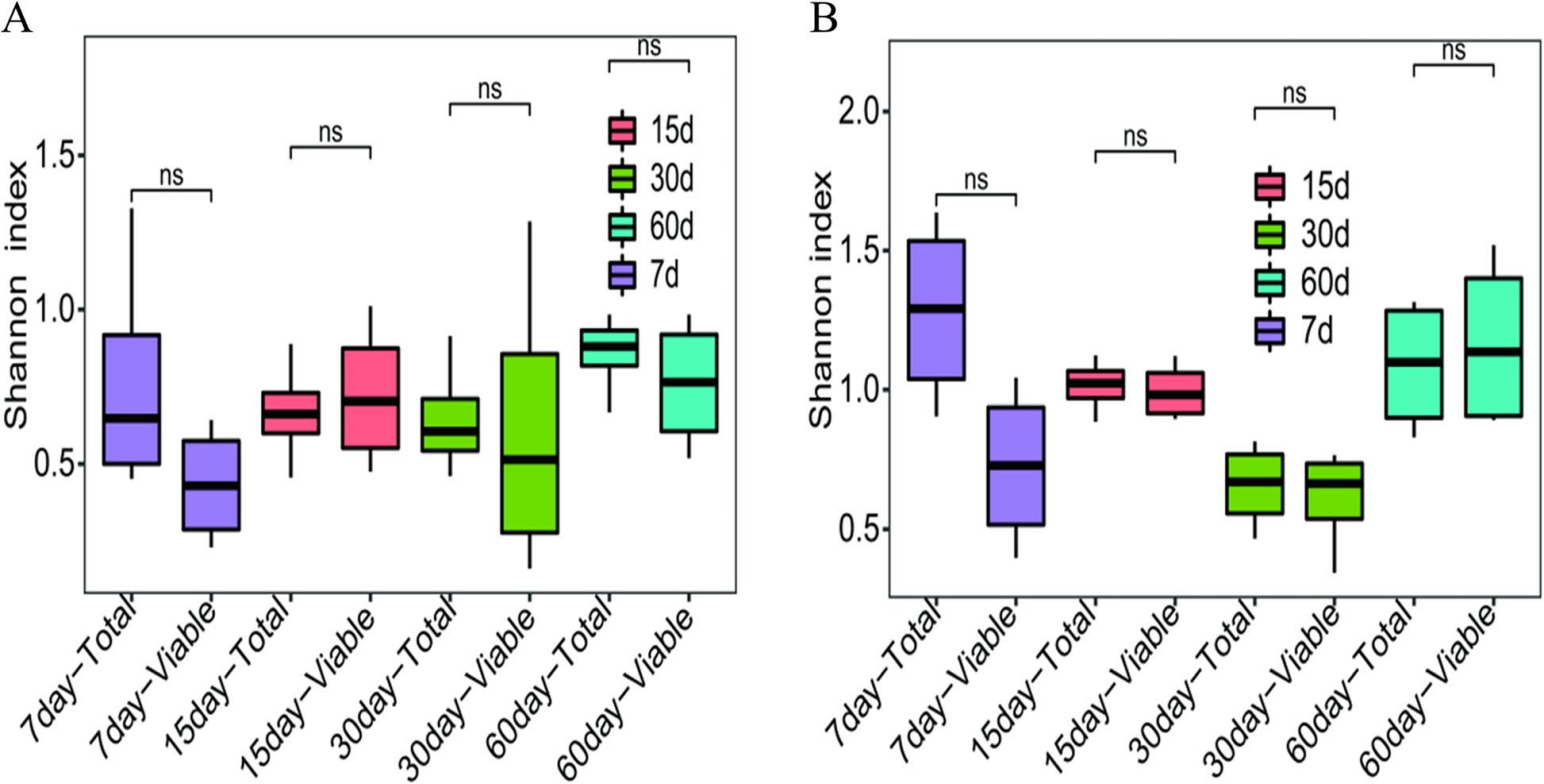
Shannon index of total and viable fungal communities in zaopei samples from pits with age of 5-year (**A**) and 20-year (**B**). The zaopei samples were treated with PMA for identifying viable fungi (Viable), whereas they were untreated with PMA for identifying total fungi (Total). The Rank sum test was used to calculate the significant difference by R software (* represents significant difference, ns represents no significant difference)

When considering Shannon index, there were no significant differences in fungal diversity between total fungi and viable fungi in zaopei from 5-year pit (Wilcoxon rank sum test, *P* > 0.05) or 20-year pit (Wilcoxon rank sum test, P > 0.05) (Fig. 6). These results indicated that non-viable fungi had little effect on the fungal community diversity during the fermentation cycle. Meanwhile, the diversity of viable fungi in zaopei from 20-year pit was notably higher than that in zaopei from 5-year pit at each fermentation time (Fig. S3), hinting that the age of pit may be a critical factor for baijiu production.

**Fig. 7:**
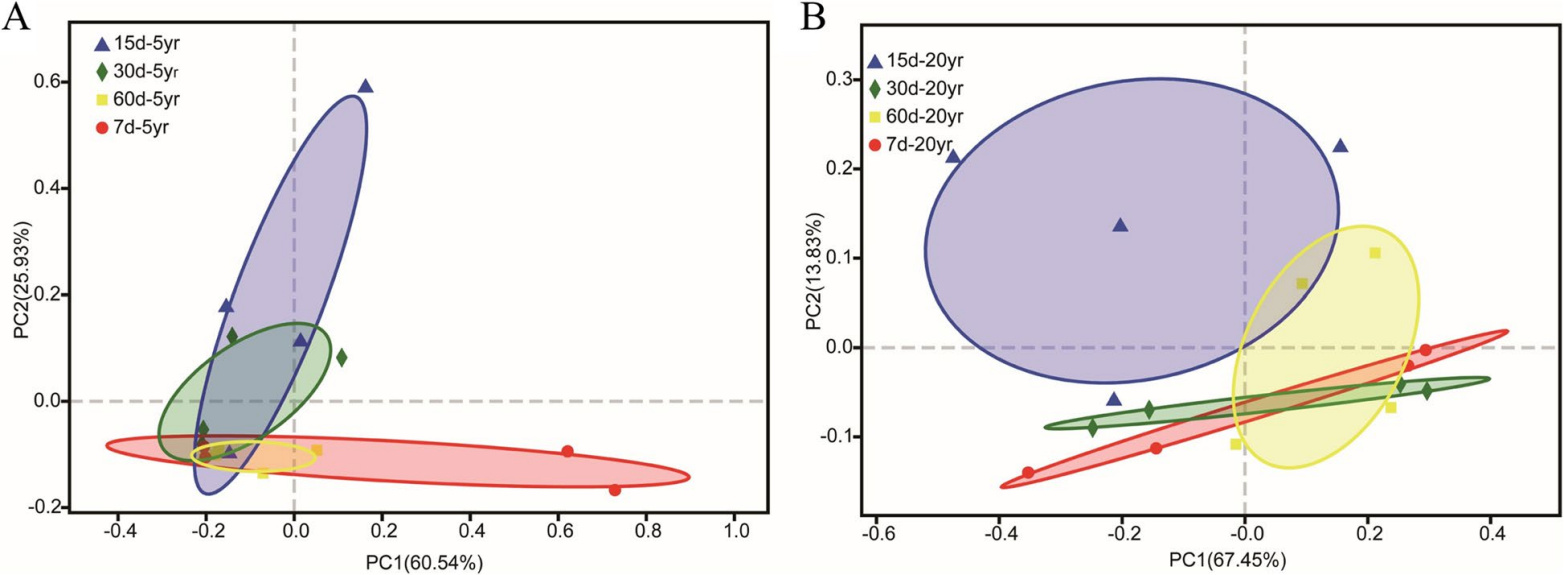
PCoA analysis of viable fungi in zaopei from 5-year pit (**A**) and 20-year pit (**B**)

PCoA based on the OTUs of ITS2 gene was performed to analyze the variation of viable fungal communities in zaopei during the fermentation cycle (Fig. 7). For the viable fungal community in zaopei from 5-year pit, the results showed the community structure of viable fungi closely overlapped among the different fermentation time (day 7, day 15, day 30, and day 60), revealing that there were no differences among the fermentation cycle from day 7 to day 60 (*p* > 0.05) (Fig. 7A and Table S2). For the viable fungal community in zaopei from 20-year pit, the results showed the community structure of viable fungi at day 15 was mostly separated from these at day 7 and day 30, hinting that there were significant differences within fermentation cycle between day 15 and day 7 (*p* < 0.05), and between day 15 and day 30 (p < 0.05) (Fig. 7B and Table S2). Herein, the variation of viable fungi in zaopei from 5-year pit differed from that of 20-year pit. The community structure of viable fungi in zaopei from 5-year pit remained stable, however, it varied significantly during the first 30 days of fermentation and then tended to stabilize in zaopei from 20-year pit.

### Composition analysis of total and viable fungi in zaopei

**Fig. 8:**
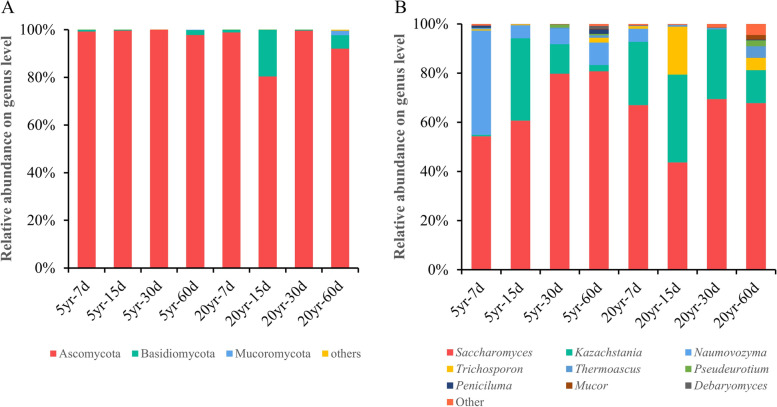
The relative abundances of viable fungi in zaopei from pit with age of 5 year and 20 year at levels of phylum (**A**), and genus (**B**). The zaopei samples were treated with PMA for identifying viable fungi. The percent of community abundance is the average value

The relative abundance of total fungi and viable fungi at different levels, including OTU, phylum, class, and genus levels, were analyzed to investigate the composition differences in zaopei samples from two kinds of pits. The results of relative abundance showed that total fungi and viable fungi displayed no significant differences at OTU, phylum, or genus levels (Fig. S4–6). However, viable fungi in zaopei are more important than total fungi in zaopei, because it elucidates the real fungal composition at a specific fermentation time. Therefore, we focused on analyzing the composition and variation of viable fungal communities during the fermentation cycle. The dominant phyla were defined as that the relative abundance of the detected phyla was higher than 1.0% in at least one zaopei sample, and the dominant genus were defined as the same standard. Herein, 3 dominant phyla and 9 dominant genera were clarified in all zaopei samples based on viable fungi analysis. The viable fungal community in zaopei from 5-year pit and 20-year pit shared similar compositions during the entire fermentation process, and Ascomycota was the most dominant viable fungus at the phylum level (Fig. 8A). At the genus level, the composition of viable fungi fluctuated with fermentation time. *Saccharomyces*, *Kazachstania*, *Naumovozyma*, *Trichosporon*, *Thermoascus*, *Pseudeurotium*, *Penicillium*, *Mucor*, and *Debaryoomyces* were the dominant viable fungi (Fig. 8B). For the viable fungi from zaopei samples in 5-year pit, genus *Saccharomyces*, *Naumovozyma* and *Kazachstania* dominated the whole fermentation cycle. During which, the relative abundance of *Saccharomyces* increased steadily from 54.30 to 80.73%, while that of *Naumovozyma* decreased from 42.40 to 9.18%. Interestingly, *Kazachstania* mainly appeared from day 15 to 30. However, the structure of viable fungi in 20-year pit was different form that in 5-year pit. Genus *Saccharomyces* and *Kazachstania* dominated the entire fermentation cycle, and the relative abundance of *Saccharomyces* remained stable at the value of 64.24% (on average), while that of *Kazachstania* stabilized at 27.02% (on average) from day 7 to day 30 then dropped to 13.43% at day 60.

## Discussion

The brewing of CSFB was performed in a pit (a special cellar below ground), and the zaopei (fermented grains) in which was fermented with a period of 60 days for producing baijiu. There are innumerable microbes involved in metabolizing raw materials to ethanol and flavor compounds. Many previous studies have investigated the change of bacterial community during fermentation process [11, 14, 15, 29], however, they only concentrated on the total bacteria and ignored the exist of non-viable bacteria and fungi. The PMA is a non-viable microbe cells dependent DNA molecular dye, and it only interacts with the DNA from the non-viable cells, prohibiting DNA amplification [20]. Thus, the PMA was adopted to treat zaopei samples for removing the non-viable microbes, then qPCR and HTS aiming at ITS2 region were performed to accurately analyze the variation and composition of viable fungi during the fermentation cycle of CSFB in this study. Meanwhile, the moisture, acidity, starch content and flavor compounds in zaopei from the two kinds of pits were measured during the whole fermentation process.

In our previous study, PMA concentration was optimized for treating pit mud samples, and a 50 μM PMA concentration was highly effective to remove the DNA from non-viable bacteria [16]. Herein, 50 μM of PMA was used and the results showed it worked effectively to remove the interference of DNA from non-viable microbe cells when the cell number lower than 2.11 × 10^13^ (Fig. 4). Moreover, adding heat-killed *E. coli* cells into zaopei suspension validated the effectiveness of PMA treatment (Fig. 4). Therefore, 50 μM was an effective concentration to treat zaopei sample with PMA for removing the interference of DNA from non-viable microbial cells, which was consistent with our previous study [16].

During the entire fermentation cycle, the starch content decreased then stabilized, and starch content in zaopei from 20-year pit was lower than that from 5-year pit (Fig. 3C). These indicated that more substrates were consumed by microbes in zaopei from older pit, implying more acid and ethanol will be produced in zaopei from the older pit. The ethanol content in zaopei from older pit reached 60%, which was higher than that in zaopei from younger pit (58%, data not shown). These results and acidity results (Fig. 3B) confirmed our previous speculation.

From the results of qPCR analysis (Fig. 5) and Shannon index (Fig. 6) on total fungi and viable fungi revealed that a very small amount of non-viable fungi existed among all zaopei samples. There were no significant differences between total fungi and viable fungi in zaopei samples during the whole fermentation cycle. During the early fermentation stage (1–15 days), the low acidity and high starch content made zaopei very suitable for the growth of fungi. The proliferation and death of fungi was equivalent, leading to that no differences were observed between total fungi and viable fungi. During the middle and late fermentation stages (15–60 days), the high acidity and low starch content provide a very hard habitat for fungi. Therefore, only the selected fungi survived at acidic and oligotrophic environment, and the DNA from non-viable fungi may be degraded quickly under the extremely acidic environment. Thus, the detectable non-viable fungi were very few during the middle and late fermentation stage (15–60 days). Also, the relationship between community structure of viable fungi and fermentation parameters (moisture, acidity, and starch) was calculated based on mantel test. The moisture, acidity and starch showed no significant (*p* > 0.05) correlations with viable fungal community (Table S3), which was similar to the results of Tan’s research [30].

When considering the composition of viable fungi, the *Saccharomyces*, *Kazachstania*, *Naumovozyma*, and *Trichosporon* were the most dominant viable fungi in zaopei, which accounted over 97.48% (zaopei from 5-year pit) and 95.38% (zaopei from 5-year pit) of viable fungi (Fig. 8). These results were partially similar with Guan’s research which reported *Saccharomyces*, *Kazachstania*, *Thermoascus*, *Aspergillus*, and *Thermomyces* were the dominant fungi [31], however, differed from others’ studies. He et al. revealed that *Aspergillus*, *Candida*, and *Thermoascus* were the dominant fungi [32], while Zhang’ s study demonstrated that *Pichia*, *Saccharomyces*, *Thermoascus* and *Aspergillus* dominated the fungal community [33]. *Zygosaccharomyces* and *Saccharomyces* were the dominant fungal community (> 95%) in the later fermentation batches [34]. *Saccharomyces* is responsible for the production of alcohol [35], and they promote the produce of flavor compounds (acetic acid, ethyl acetate, and ethyl caproate) by the conversion of acetyl-CoA during fermentation [36–39]. Interestingly, *Naumovozyma* was firstly detected in zaopei samples from 5-year pit with a trend of decrease from 42.40 to 9.18%, however, it was replaced by *Kazachstania* in zaopei samples from 20-year pit. *Naumovozyma* and *Kazachstania* are belong to yeast strains, suggesting that they played a role in producing ethanol during baijiu fermentation. Furthermore, *Kazachstania* was reported to make a positive contribution to wine aroma by producing ethyl acetate, ethyl lactate and acetic acid [40], while the function of *Naumovozyma* in zaopei was unknown. Therefore, the function of *Naumovozyma* and *Kazachstania* in zaopei from CSFB needs further study.

It was previously reported that esters are the foundation of flavor in baijiu, especially ethyl caproate, which is the key aroma substance of CSFB. The content of ethyl caproate is positively correlated to the quality of CSFB [41], and the content of ethyl lactate have an influence on the quality of CSFB, too [3]. Here, the content of ethyl caproate increased significantly during the entire fermentation period (Fig. 3I). The content of ethyl caproate in zaopei from 20-year pit was twice that from 5-year pit, implying the quality of baijiu produced from older pit will be better than that produced from younger pit. Besides esters, organic acids play a vital role on the quality of baijiu, mainly changing the taste of baijiu [2, 13]. It was found that acidity, acetic acid, lactic acid and butyric acid increased with the prolongation of fermentation period (Fig. 2B, D, E, and F). This result hints that the whole fermentation time should not be too short (less than 30 days) in the production of CSFB. Because esters and organic acids were mainly produced by microorganisms in zaopei during the middle and late fermentation (from day 15 to day 60), and appropriately prolonging the fermentation period will be conducive to enhancing the quality of CSFB, which was consistent with Zheng’s study [42].

## Conclusion

In this study, we firstly used PMA to treat zaopei samples from two kinds of pits for removing the interference of non-viable fungi, and analyzed the diversity of viable fungi. The 50 μM of PMA can effectively remove the interference of DNA from non-viable microbial cells. Results showed that non-viable fungi have little effect on the fungal diversity, structure, and relative abundance in zaopei samples, and there were no significant differences between total fungi and viable fungi in two kinds of zaopei samples during the whole fermentation cycle. A total of 6 phyla, 19 classes, and 118 genera in fungi were identified based on OTUs annotation. It was found that the most dominant viable fungi belonged to *Saccharomyces*, *Kazachstania*, *Naumovozyma*, and *Trichosporon*, and *Naumovozyma* was firstly detected in two kinds of zaopei samples of CSFB. Moreover, the quality of CSFB produced from older pit was better than that produced from younger pit. Our findings can be applied as guidance for improving the quality and stability of CSFB.

## Supplementary Information

**Additional file 1: Fig. S1** Schematic diagram of PMA action process. **Fig. S2**. The variation of viable fungi detected by qPCR in zaopei samples from pits with age of 5-year and 20-year. The one-way ANOVA test was used to compare the differences between two pits. **Fig. S3**. Shannon index changes of viable fungal community in zaopei samples from pits with age of 5-year and 20-year. The one-way ANOVA test was used to compare the differences between two pits. **Fig. S4**. Statistically significant differences in the relative abundance of the top 15 OTU between total fungi and viable fungi in zaopei from 5-year pit (A, B, C, and D) and 20-year pit (E, F, G, and H) at different fermentation time. **Fig. S5**. Statistically significant differences in the relative abundance of all the phyla between total fungi and viable fungi in zaopei from 5-year pit (A, B, C, and D) and 20-year pit (E, F, G, and H) at different fermentation time. Fig. S6. Statistically significant differences in the relative abundance of the top 15 genera between total fungi and viable fungi in zaopei from 5-year pit (A, B, C, and D) and 20-year pit (E, F, G, and H) at different fermentation time. **Table S1**. Read number and sequencing average of ITS2 sequencing in zaopei. **Table S2** Permanova analysis of viable fungal community during fermentation process. **Table S3** Mantel test analysis between viable fungi in fermented grains and fermentation parameters.

## Data Availability

All data generated or analyzed during this study are included in this published article and its Additional file.

## Abbreviations

CSFB: Chinese strong-flavor baijiu
PMA: Propidium monoazide
qPCR: Quantitative polymerase chain reaction
HTS: High-throughput sequencing
ITS: Internal transcribed spacer
OTU: Operational taxonomic unit
PCR-DGGE: Polymerase chain reaction-Denaturing gradient gel electrophoresis
PCoA: Principal coordinates analysis
