# Supplementary material for: Detection of viable and total fungal community in zaopei of Chinese strong-flavor baijiu using PMA combined with qPCR and HTS based on ITS2 region
Source: BMC Microbiol. 2021 Oct 8;21:274. doi: 10.1186/s12866-021-02334-8 (PMC8499482; doi:10.1186/s12866-021-02334-8)
Supplement: Supplementary file 1 — Additional file 1: Fig. S1 Schematic diagram of PMA action process. Fig. S2. The variation of viable fungi detected by qPCR in zaopei samples from pits with age of 5-year and 20-year. The one-way ANOVA test was used to compare the differences between two pits. Fig. S3. Shannon index changes of viable fungal community in zaopei samples from pits with age of 5-year and 20-year. The one-way ANOVA test was used to compare the differences between two pits. Fig. S4. Statistically significant differences in the relative abundance of the top 15 OTU between total fungi and viable fungi in zaopei from 5-year pit (A, B, C, and D) and 20-year pit (E, F, G, and H) at different fermentation time. Fig. S5. Statistically significant differences in the relative abundance of all the phyla between total fungi and viable fungi in zaopei from 5-year pit (A, B, C, and D) and 20-year pit (E, F, G, and H) at different fermentation time. Fig. S6. Statistically significant differences in the relative abundance of the top 15 genera between total fungi and viable fungi in zaopei from 5-year pit (A, B, C, and D) and 20-year pit (E, F, G, and H) at different fermentation time. Table S1. Read number and sequencing average of ITS2 sequencing in zaopei. Table S2 Permanova analysis of viable fungal community during fermentation process. Table S3 Mantel test analysis between viable fungi in fermented grains and fermentation parameters. [file 12866_2021_2334_MOESM1_ESM.docx]

**Supplementary material for**

**Detection of viable and total fungal community in zaopei of Chinese strong-flavor baijiu using PMA combined with qPCR and HTS based on ITS2 region**

Huanming Liu^1,2^, Guangxun Tan^1,3^, Qitong Chen^1^, Weiwei Dong^1^, Ping Chen^3^, Kaiyun Cai^3^, Yuanliang Hu^4^, Weiyan Zhang^5^, Nan Peng^1^, Yunxiang Liang^1^, Shumiao Zhao^1^*

^1^ State Key Laboratory of Agricultural Microbiology and College of Life Science and Technology, Huazhong Agricultural University, Wuhan 430070, China.

^2^ School of Food Science and Technology, Guangdong Ocean University, Zhanjiang 524088, China.

^3^ Hubei Daohuaxiang Liquor Co., Ltd, Yichang 443112, China.

^4^ Hubei Key Laboratory of Edible Wild Plants Conservation＆Utilization, College of Life Sciences, Hubei Normal University, Huangshi 435000, China.

​^5^ Li Dak Sum Yip Yio Chin Kenneth Li Marine Biopharmaceutical Research Center, Ningbo University, Ningbo 315211, China.

***Corresponding authors:**

Shumiao Zhao, Tel. /fax: +8602787281040;

E-mail address: shumiaozhao@mail.hzau.edu.cn


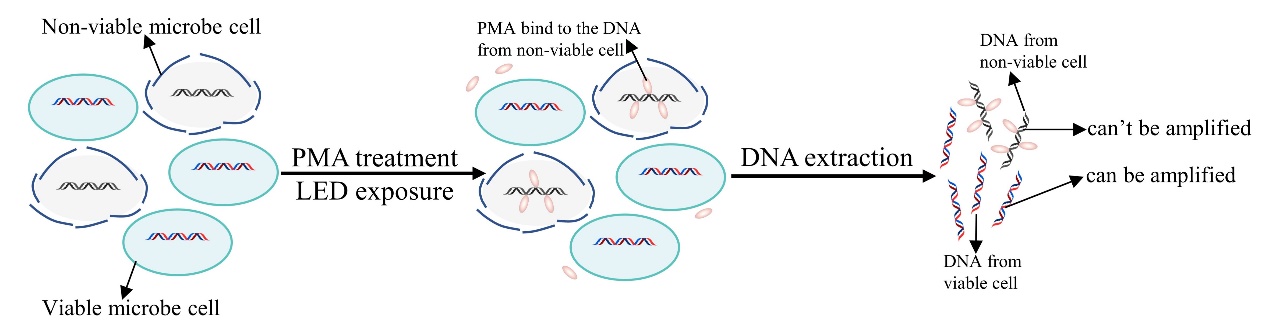


Fig. S1 Schematic diagram of PMA action process.


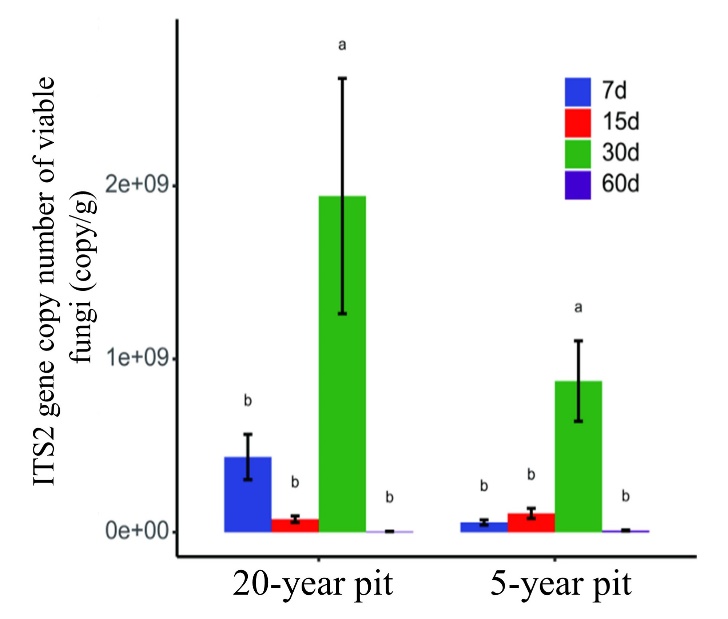


Fig. S2. The variation of viable fungi detected by qPCR in zaopei samples from pits with age of 5-year and 20-year. The one-way ANOVA test was used to compare the differences between two pits.


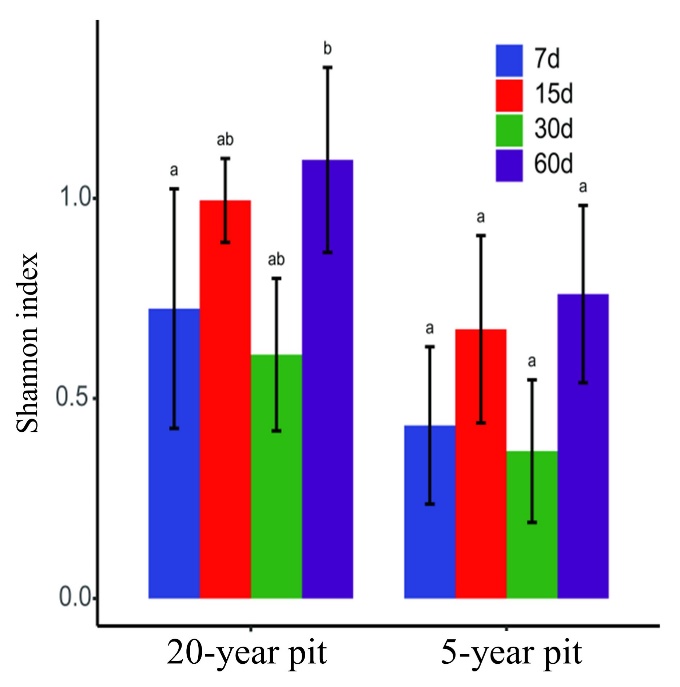


Fig. S3. Shannon index changes of viable fungal community in zaopei samples from pits with age of 5-year and 20-year. The one-way ANOVA test was used to compare the differences between two pits.


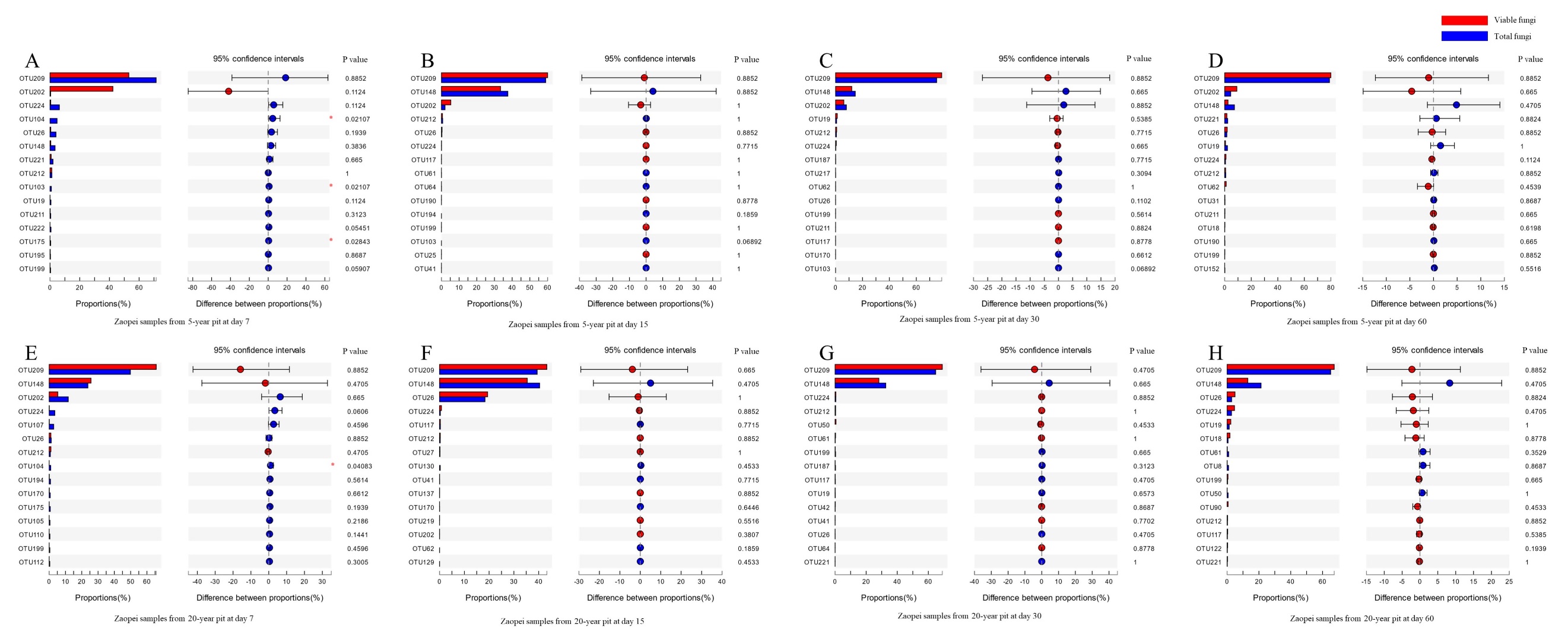


Fig. S4. Statistically significant differences in the relative abundance of the top 15 OTU between total fungi and viable fungi in zaopei from 5-year pit (A, B, C, and D) and 20-year pit (E, F, G, and H) at different fermentation time.


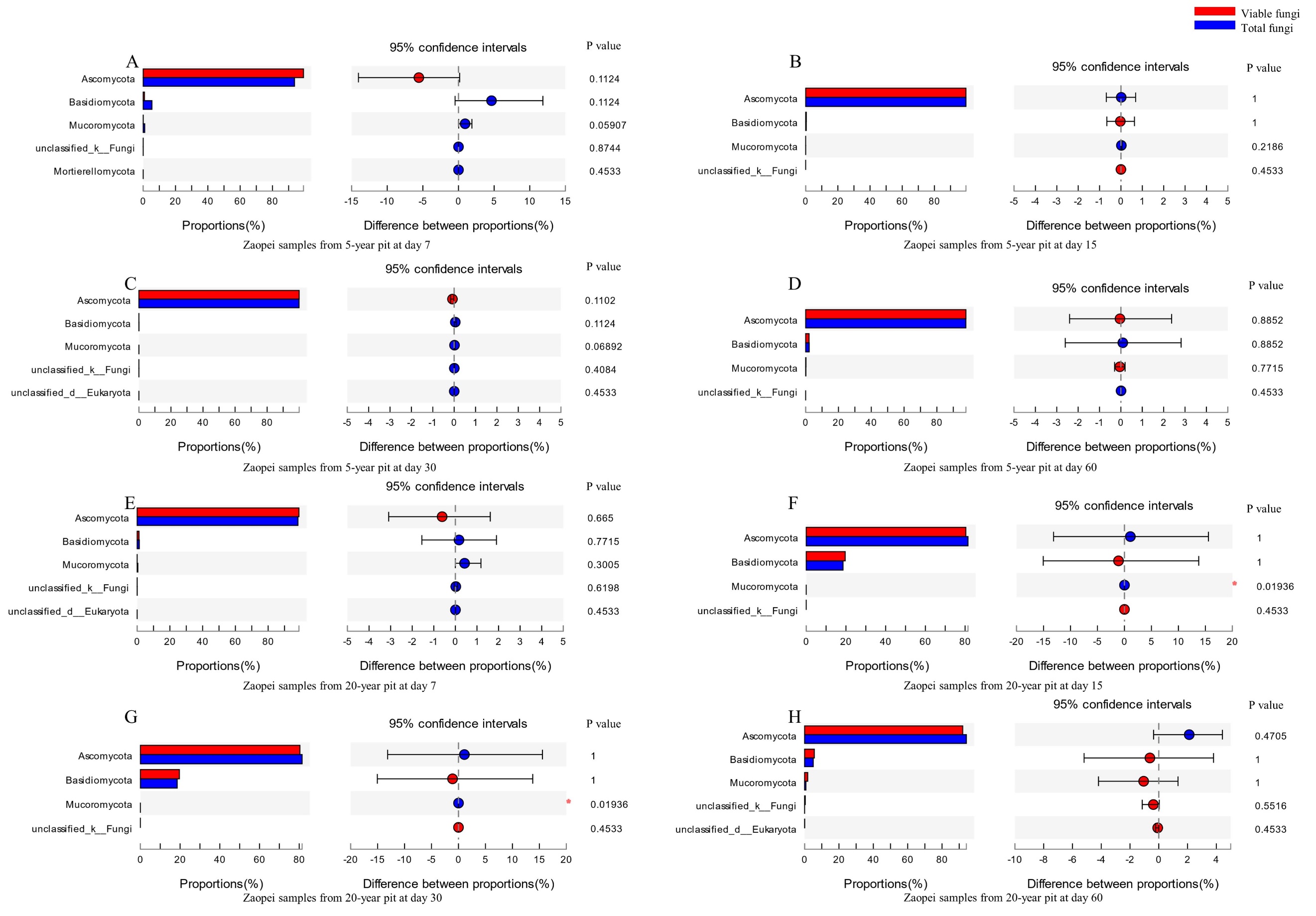


Fig. S5. Statistically significant differences in the relative abundance of all the phyla between total fungi and viable fungi in zaopei from 5-year pit (A, B, C, and D) and 20-year pit (E, F, G, and H) at different fermentation time.


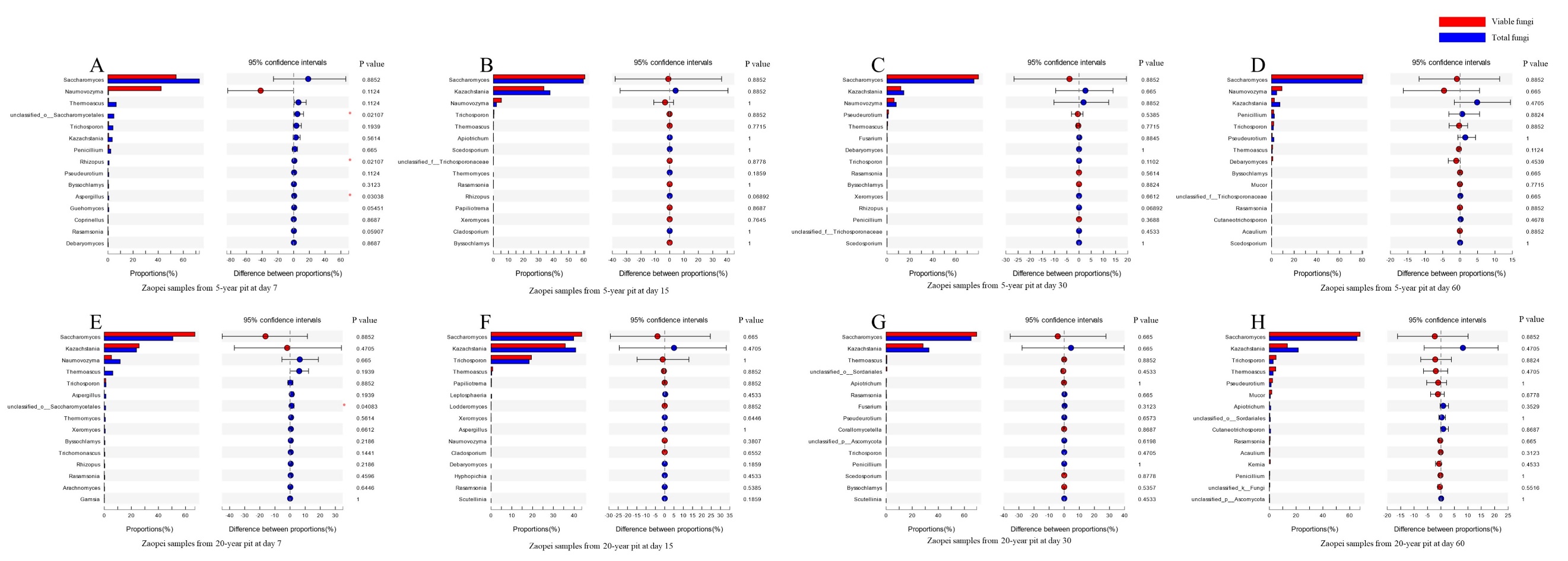


Fig. S6. Statistically significant differences in the relative abundance of the top 15 genera between total fungi and viable fungi in zaopei from 5-year pit (A, B, C, and D) and 20-year pit (E, F, G, and H) at different fermentation time.

Table S1. Read number and sequencing average of ITS2 sequencing in zaopei

| Sample | Seq num | Mean length | Sample | Seq num | Mean length |
| --- | --- | --- | --- | --- | --- |
| 5year-7day-Total-1 | 44843 | 285.97 | 20year-7day-Total-1 | 47655 | 319.15 |
| 5year-7day-Total-2 | 44845 | 320.17 | 20year-7day-Total-2 | 34346 | 305.16 |
| 5year-7day-Total-3 | 38101 | 323.32 | 20year-7day-Total-3 | 48762 | 323.56 |
| 5year-7day-Total-4 | 49972 | 324.91 | 20year-7day-Total-4 | 33219 | 325.22 |
| 5year-7day-Viable-1 | 38702 | 332.05 | 20year-7day-Viable-1 | 50802 | 324.54 |
| 5year-7day-Viable-2 | 43313 | 336.13 | 20year-7day-Viable-2 | 43151 | 323.98 |
| 5year-7day-Viable-3 | 43958 | 326.53 | 20year-7day-Viable-3 | 53949 | 323.74 |
| 5year-7day-Viable-4 | 50251 | 326.47 | 20year-7day-Viable-4 | 36463 | 326.03 |
| 5year-15day-Total-1 | 41020 | 324.56 | 20year-15day-Total-1 | 74216 | 309.47 |
| 5year-15day-Total-2 | 62385 | 321.50 | 20year-15day-Total-2 | 55051 | 312.22 |
| 5year-15day-Total-3 | 32172 | 324.66 | 20year-15day-Total-3 | 47516 | 307.83 |
| 5year-15day-Total-4 | 33587 | 324.92 | 20year-15day-Total-4 | 31340 | 318.98 |
| 5year-15day-Viable-1 | 51469 | 325.91 | 20year-15day-Viable-1 | 72919 | 302.85 |
| 5year-15day-Viable-2 | 49622 | 321.24 | 20year-15day-Viable-2 | 70418 | 311.73 |
| 5year-15day-Viable-3 | 42573 | 324.39 | 20year-15day-Viable-3 | 39035 | 311.16 |
| 5year-15day-Viable-4 | 42251 | 326.90 | 20year-15day-Viable-4 | 44924 | 321.73 |
| 5year-30day-Total-1 | 55482 | 327.59 | 20year-30day-Total-1 | 59264 | 323.25 |
| 5year-30day-Total-2 | 54788 | 323.88 | 20year-30day-Total-2 | 52760 | 323.62 |
| 5year-30day-Total-3 | 39180 | 324.82 | 20year-30day-Total-3 | 35834 | 321.82 |
| 5year-30day-Total-4 | 35475 | 325.39 | 20year-30day-Total-4 | 45560 | 325.68 |
| 5year-30day-Viable-1 | 33546 | 326.14 | 20year-30day-Viable-1 | 33853 | 323.83 |
| 5year-30day-Viable-2 | 60299 | 322.58 | 20year-30day-Viable-2 | 62607 | 321.51 |
| 5year-30day-Viable-3 | 34119 | 324.91 | 20year-30day-Viable-3 | 46224 | 322.90 |
| 5year-30day-Viable-4 | 41989 | 326.18 | 20year-30day-Viable-4 | 38795 | 326.28 |
| 5year-60day-Total-1 | 61763 | 320.45 | 20year-60day-Total-1 | 61326 | 314.79 |
| 5year-60day-Total-2 | 35742 | 322.09 | 20year-60day-Total-2 | 66545 | 312.19 |
| 5year-60day-Total-3 | 42875 | 324.22 | 20year-60day-Total-3 | 39245 | 322.38 |
| 5year-60day-Total-4 | 49489 | 316.86 | 20year-60day-Total-4 | 44931 | 320.46 |
| 5year-60day-Viable-1 | 47588 | 322.19 | 20year-60day-Viable-1 | 70093 | 311.07 |
| 5year-60day-Viable-2 | 49073 | 327.09 | 20year-60day-Viable-2 | 63527 | 310.15 |
| 5year-60day-Viable-3 | 44902 | 321.83 | 20year-60day-Viable-3 | 37726 | 317.85 |
| 5year-60day-Viable-4 | 37566 | 320.46 | 20year-60day-Viable-4 | 45161 | 318.87 |

Table S2 Permanova analysis of viable fungal community during fermentation process

| Pit age | pairs | R2 | p.value | sig |
| --- | --- | --- | --- | --- |
| 5-year | 7day vs 15day | 0.041 | 0.246 |  |
|  | 7day vs 30day | 0.152 | 0.912 |  |
|  | 7day vs 60day | 0.277 | 1.000 |  |
|  | 15day vs 30day | 0.081 | 0.486 |  |
|  | 15day vs 60day | 0.005 | 0.030 |  |
|  | 30day vs 60day | 0.184 | 1.000 |  |
| 20-year | 7day vs 15day | 0.346 | 0.007 | * |
|  | 7day vs 30day | 0.012 | 0.933 |  |
|  | 7day vs 60day | 0.040 | 0.553 |  |
|  | 15day vs 30day | 0.516 | 0.001 | * |
|  | 15day vs 60day | 0.354 | 0.004 | * |
|  | 30day vs 60day | 0.102 | 0.222 |  |

Table S3 Mantel test analysis between viable fungi in fermented grains and fermentation parameters

| Fungi | 7day |  | 15day |  | 30day |  | 60day |  |
| --- | --- | --- | --- | --- | --- | --- | --- | --- |
|  | R | p | R | p | R | p | R | p |
| Moisture | -0.188 | 0.848 | -0.288 | 0.916 | -0.22 | 0.893 | 0.179 | 0.236 |
| Titratable acidity | -0.318 | 0.981 | -0.189 | 0.762 | -2.036 | 0.897 | 0.024 | 0.428 |
| Starch | -0.125 | 0.681 | 0.111 | 0.255 | -0.234 | 0.976 | 0.169 | 0.178 |
